# Maize (*Zea mays* L.) Genome Diversity as Revealed by RNA-Sequencing

**DOI:** 10.1371/journal.pone.0033071

**Published:** 2012-03-16

**Authors:** Candice N. Hansey, Brieanne Vaillancourt, Rajandeep S. Sekhon, Natalia de Leon, Shawn M. Kaeppler, C. Robin Buell

**Affiliations:** 1 Department of Plant Biology, Michigan State University, East Lansing, Michigan, United States of America; 2 Department of Energy Great Lakes Bioenergy Research Center, Michigan State University, East Lansing, Michigan, United States of America; 3 Department of Agronomy, University of Wisconsin-Madison, Madison, Wisconsin, United States of America; 4 Department of Energy Great Lakes Bioenergy Research Center, University of Wisconsin-Madison, Madison, Wisconsin, United States of America; American University in Cairo, Egypt

## Abstract

Maize is rich in genetic and phenotypic diversity. Understanding the sequence, structural, and expression variation that contributes to phenotypic diversity would facilitate more efficient varietal improvement. RNA based sequencing (RNA-seq) is a powerful approach for transcriptional analysis, assessing sequence variation, and identifying novel transcript sequences, particularly in large, complex, repetitive genomes such as maize. In this study, we sequenced RNA from whole seedlings of 21 maize inbred lines representing diverse North American and exotic germplasm. Single nucleotide polymorphism (SNP) detection identified 351,710 polymorphic loci distributed throughout the genome covering 22,830 annotated genes. Tight clustering of two distinct heterotic groups and exotic lines was evident using these SNPs as genetic markers. Transcript abundance analysis revealed minimal variation in the total number of genes expressed across these 21 lines (57.1% to 66.0%). However, the transcribed gene set among the 21 lines varied, with 48.7% expressed in all of the lines, 27.9% expressed in one to 20 lines, and 23.4% expressed in none of the lines. *De novo* assembly of RNA-seq reads that did not map to the reference B73 genome sequence revealed 1,321 high confidence novel transcripts, of which, 564 loci were present in all 21 lines, including B73, and 757 loci were restricted to a subset of the lines. RT-PCR validation demonstrated 87.5% concordance with the computational prediction of these expressed novel transcripts. Intriguingly, 145 of the novel *de novo* assembled loci were present in lines from only one of the two heterotic groups consistent with the hypothesis that, in addition to sequence polymorphisms and transcript abundance, transcript presence/absence variation is present and, thereby, may be a mechanism contributing to the genetic basis of heterosis.

## Introduction

Maize is genetically and phenotypically diverse. In populations containing only a small proportion of the variation present in the entirety of maize, progress based on phenotypic selection is being realized. This is exemplified by consistent gains in hybrid yields [1], [2] and through progress in long-term selection experiments, such as the Illinois long-term selection study for grain oil and protein content [3], selection for prolificacy [4], and selection for seed size [5], [6]. Phenotypic diversity in maize for yield, composition, and morphological traits has also been documented in multiple diversity panels [7], [8], [9], and in analysis of structured populations such as the nested association mapping (NAM) population, which represents diverse maize types [10], [11], [12], [13], [14]. Understanding the genetic factors that underlie this extensive phenotypic diversity and allow for continual improvement in populations is essential for efficient manipulation of maize to meet the demands of the increasing human population and the need to adapt to global climate changes. 

A wide range of sequence level variation exists in maize including single nucleotide polymorphisms (SNPs), small insertions/deletions, presence/absence variation (PAV), and copy number variation (CNV) [15], [16], [17], [18], [19]. Furthermore, epigenetic mechanisms also generate variation via differences in transcript abundance [16], [18], [19], [20], [21]. With the recent release of the maize B73 reference genome [17] and the availability of high-output, affordable sequencing technologies, a greater understanding of this diversity, particularly structural variation, is being realized.

The concept of separate core and dispensable portions of genomes was first described in prokaryotes. The core genome is defined as the portion present in all sequenced strains and the dispensable genome is defined as sequence that is present in one or more but not all strains [22], [23]. This phenomenon has been subsequently observed in many bacterial species [23], [24], [25]. In addition to understanding environmental adaptation, characterizing microbial pan-genomes has led to the development of universal vaccine cocktails using genes from the dispensable genome [26], [27], the elucidation of virulence factors [28], and improved pathogen diagnostics [29]. The presence of a pan-genome has also been demonstrated in eukaroyote species, including human (*Homo sapiens*) [30], maize (*Zea mays* L.) [16], [18], [19], [31], [32], and *Arabidopsis thaliana* [33], [34], [35]; however, the size and effect of the dispensable genome in eukaryotes is not well understood. 

The goal of this study was to explore sequence and expression-based variation, and to identify novel transcripts in a common set of diverse maize lines using RNA based sequencing (RNA-seq). RNA-seq permits all of these types of variation to be evaluated simultaneously in a cost effective manner. It also has a major advantage in large complex genomes of reducing the effective genome size substantially. For example, the 2.3 Gb haploid maize genome is reduced to a 97 Mb transcriptome assuming all genes are expressed. In this study, we generated 17.8 Gb of seedling RNA-seq data to explore a set of 21 diverse maize lines that are representative of North American as well as exotic germplasm to improve our understanding of the maize genome and the genetic relationships between lines with diverse pedigrees.

## Results and Discussion

### Description of Germplasm and Datasets

The inbred lines used in this study were selected to provide a representative sampling of genetic and phenotypic variation in maize [7], [8], [15], [16], [17], [18], [19] by inclusion of diverse exotic germplasm, and germplasm from two major heterotic groups involved in United States grain hybrids, Stiff Stalk Synthetic (SSS) and Non-Stiff Stalk Synthetic (NSS) [36]. We generated between 6.2 and 39.7 million single end (36–74 bp) RNA-seq reads for each of the 21 inbred lines (Table 1). This dataset allows for in-depth evaluation of sequence and expression variation, as well as the identification of novel expressed maize sequences in a common set of 21 maize lines.

**Table 1:** Read mapping, expression, and single nucleotide polymorphism (SNP) summary for 21 diverse maize lines.

Inbred Line	Group	Purity Filtered Reads	% Reads Mapped FPKM	% Reads Mapped SNP	% Reads Mapped with Assembled Transcripts	Max FPKM	% Genes Expressed	% SNPs with Coverage	% Genes with SNP Coverage
B14A	SSS	11.1M	77.85%	57.79%	78.34%	37,727	61.48%	64.8%	50.0%
B37	SSS	20.5M	82.65%	61.07%	83.19%	36,901	64.73%	81.3%	53.7%
B73	SSS	19.4M	87.81%	64.17%	88.31%	37,978	64.51%	83.9%	53.1%
B97	NSS	19.7M	82.00%	60.07%	82.57%	41,010	64.17%	79.8%	53.3%
CML103	Exotic	10.0M	80.47%	58.01%	81.05%	40,148	61.02%	63.9%	49.4%
CML322	Exotic	20.7M	82.10%	59.44%	82.64%	40,302	64.69%	79.5%	49.4%
CML333	Exotic	8.3M	77.80%	55.30%	78.34%	49,670	60.23%	49.8%	46.3%
H99	NA	19.7M	80.55%	59.74%	81.10%	32,713	65.05%	76.2%	53.3%
M37W	NA	17.4M	83.10%	59.76%	83.71%	50,022	63.49%	75.7%	52.2%
Mo17	NSS	9.3M	74.57%	56.35%	75.10%	27,418	61.35%	60.7%	49.6%
MoG	NA	14.8M	77.32%	57.31%	77.87%	40,860	62.91%	69.8%	51.6%
MS71	NSS	6.2M	78.07%	55.88%	78.69%	26,667	60.11%	45.9%	45.7%
NC350	Exotic	7.2M	81.50%	60.99%	82.05%	34,546	57.08%	61.1%	48.9%
NC358	Exotic	17.9M	78.61%	58.44%	79.14%	45,634	63.84%	75.0%	52.1%
Oh43	NSS	39.7M	82.84%	57.89%	83.48%	48,470	64.39%	86.0%	54.4%
Oh7B	NA	13.5M	83.38%	60.60%	83.94%	32,622	64.28%	73.1%	52.2%
PHG47	NSS	11.5M	75.77%	56.40%	76.30%	35,519	62.28%	64.3%	50.4%
PHN11	Iodent	10.7M	80.47%	57.42%	81.10%	34,790	61.76%	66.1%	50.2%
PHW65	NSS	19.1M	83.42%	59.80%	83.99%	53,606	64.44%	78.6%	53.1%
W605S	NSS	6.4M	78.48%	55.61%	79.12%	28,518	59.12%	45.2%	45.0%
W64A	NSS	29.5M	80.67%	59.94%	81.24%	39,564	65.96%	86.3%	54.6%

Reads were mapped requiring a unique hit for the SNPs and multiple hits for fragments per kilobase of exon model per million fragments mapped (FPKM) and mapping to the pseudomolecules plus the Velvet and Oases [45] assembled transcripts. FPKM values were calculated using Cufflinks [56]. Genes with a FPKM 95% confidence interval lower boundary greater than zero were considered expressed. For each inbred line, a gene was considered to have SNP coverage if there was at least one polymorphic locus with coverage in the gene. SSS = Stiff Stalk Synthetic, NSS = Non-Stiff Stalk Synthetic, NA = Not applicable.

Using the maize B73 5b pseudomolecules (http://ftp.maizesequence.org/), between 74.57% and 87.81% of the reads could be mapped to the reference sequence with B73 having the highest percentage of reads mapped (Table 1). The range in percent of sequences aligning to the reference sequence is reflective of the diversity of the germplasm explored in this study relative to the reference sequence. While there is a broad range in percentage of mapped reads across the 20 non-B73 lines (Table 1), for all genotypes there is still a relatively high and substantial percentage of reads mapping. In addition, the consistent proportions of reads that mapped uniquely and to multiple locations (Figure S1), suggests little bias attributable to use of the maize B73 reference sequence in downstream identification of sequence, expression, and novel expressed sequence variants among the other 20 lines. 

### Single Nucleotide Polymorphism Variation

There are limitations to studying genetic diversity using RNA-seq rather than genomic sequencing, namely that a gene/allele must be expressed in the tissue being sequenced in order to detect variants. Because the germplasm in this study is nearly homozygous, we do not expect multiple alleles at any given locus within an inbred line. Using 511 SNP makers from an Illumina Golden Gate SNP assay, the range in observed heterozygosity across these 21 lines ranged from 0.0% to 0.4% [8]. Thus, bias due to allele specific expression is not a concern. In addition, a previous study, which included 60 maize tissue types, showed that seedling tissue had the highest number of expressed genes [37]. Thus, use of seedling RNA-seq will allow for identification of a large portion of the total SNPs in the maize transcriptome. 

A major advantage of RNA-seq in SNP calling relative to whole genome sequencing is the reduction in the effective size of the genome. This reduction in effective genome size can also be accomplished with exome capture and subsequent sequencing [38]. Exome capture is advantageous in allowing for the detection of unexpressed alleles in heterozygous species as well as SNP detection in unexpressed genes. However, exome capture does not allow for simultaneous quantification of transcript abundance, another major advantage of RNA-seq. Using RNA-seq we were able to identify 351,710 high confidence SNP polymorphisms between this set of 21 diverse inbred lines (Table S1), of which, 329,027 SNPs were within annotated gene models in the version 5b annotation (http://ftp.maizesequence.org/). Congruent SNP positions with sequence coverage in at least 15 of the inbred lines were abundant within our dataset (197,720 SNPs in 17,149 genes; Table S2). Utilization of RNA-seq requires much less sequence depth to identify the majority of the variants in medium to highly expressed transcripts relative to whole genome sequencing. Without the reduction in effective genome size afforded by RNA-seq, significantly more reads or imputation following skim sequencing of the whole genome to permit consistent comparison of congruent positions across the genotypes would be required [15], [39].

To assess the error rate associated with the RNA-seq based SNP calls, genotype scores were compared to an independent SNP data set from this same set of 21 inbred lines obtained by Illumina sequencing of genomic DNA using a modified version of the genotyping-by-sequencing (GBS) protocol [39]. The B73 sequences from the RNA-seq and GBS datasets were first compared to the B73 5b reference pseudomolecules. From the RNA-seq data, 32,341,048 nucleotide positions were compared to the 2.3 Gb reference sequence, with 99.86% of the loci concordant to the reference sequence. Similarly, for the GBS data, of the 47,727,306 nucleotides compared, 99.92% matched the reference sequence. This error rate may be slightly inflated due to errors in the B73 reference sequence; however, for the 6,202,079 positions in common between the RNA-seq and GBS datasets, only 147 were different from the reference sequence and concordant between the two sequencing methods. This suggests a low error rate within the reference B73 genome sequence. To further test the quality of these datasets, data from both sequencing methods were compared across all 21 lines. Between the RNA-seq and GBS methods, there were 147,857 data points (nucleotide position-by-inbred line) in common representing 16,915 positions in the reference sequence. Of these data points, 97.25% (143,796 SNPs) were concordant between the two methods, with only 40 of the 16,915 positions having more than two inbred lines with contradicting nucleotides. Both of these comparisons indicate a very low error rate in the SNP calling methods used in this study and provide strong support for the quality of this data as well as the quality of the 5b maize reference pseudomolecule sequences.

**Figure 1:**
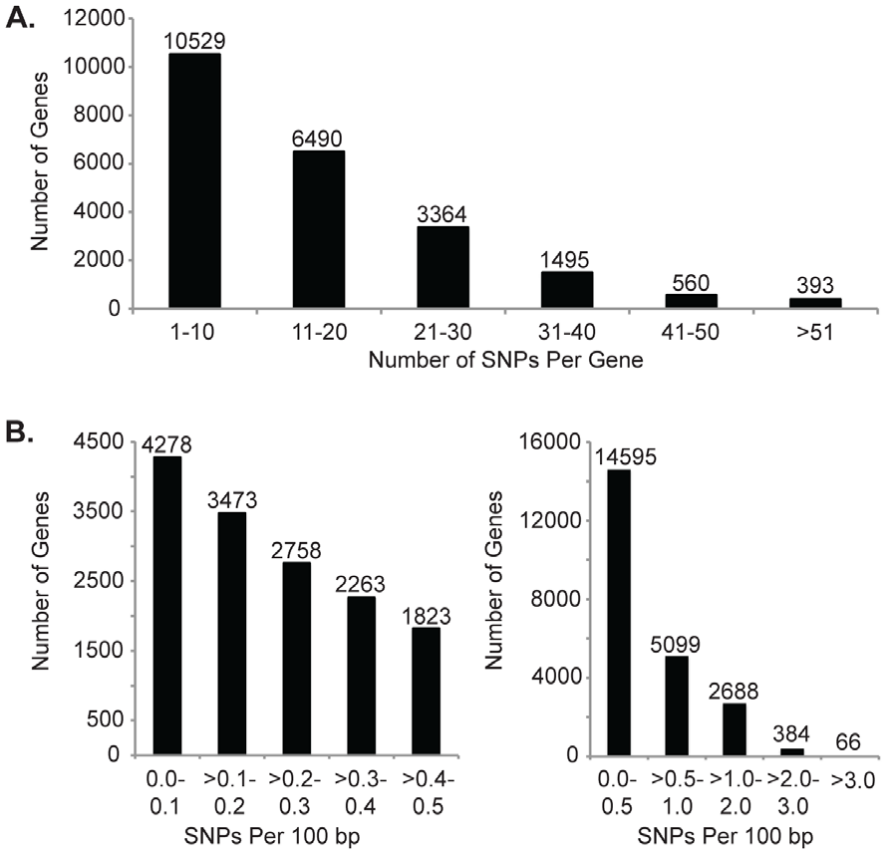
Distribution of the number of single nucleotide polymorphisms (SNPs) and SNP density per gene. Reads were mapped against the 5b pseudomolecules (http://ftp.maizesequence.org/) with Bowtie version 0.12.7 [50] and TopHat version 1.2.0 [51] requiring a unique hit for the SNP mapping. Gene assignment was determined based on the 5b annotation (http://ftp.maizesequence.org/), and not all SNPs identified were assigned to a gene model. (A) Distribution of the number of SNPs per gene. (B) Distribution of the average number of SNPs per 100 bp window per gene.

The 350,710 SNPs identified in this study were distributed throughout the genome, with the number of SNPs per 1 Mb window coincidental with gene density and number of expressed genes per window. There were, however, windows with relatively high or low SNP density compared to the number of genes and expressed genes, such as on the long arm of chromosome 2 (Figure S2). On a single gene basis, RNA-seq-derived SNPs ranged from zero SNPs to a maximum of 170 SNPs per gene, with 22,831 genes having at least one SNP (Figure 1A).

After normalizing the number of SNPs per gene for length of the gene, less than 0.5 SNPs per 100 bp were identified for the majority of the genes expressed in seedling tissue (Figure 1B). Based on a study that analyzed more than 32 Gb of sequence in the low copy region of the genome across 27 diverse lines, maize haplotypes are comprised of over 3 million SNPs and insertions/deletions with a polymorphism on average every 44 bp [15]. This discrepancy in observed SNP density is likely due to the inclusion of non-coding sequence in the genomic sequencing, which is less conserved than genic regions, consistent with observations from whole-genome sequencing of rice inbred lines [40]. Genomic sequencing of six elite Chinese inbred lines identified 468,966 SNPs in the 97 Mb gene space, averaging to approximately one SNP every 207 bp [16]. This is similar to the density observed in this study and consistent with the observation of decreased diversity in the coding sequence. There were, however, a subset of the genes with high SNP density after normalizing for gene length, with 384 genes containing 2–3 SNPs per 100 bp and 66 genes with greater than 3 SNPs per 100 bp (Figure 1B).

**Figure 2:**
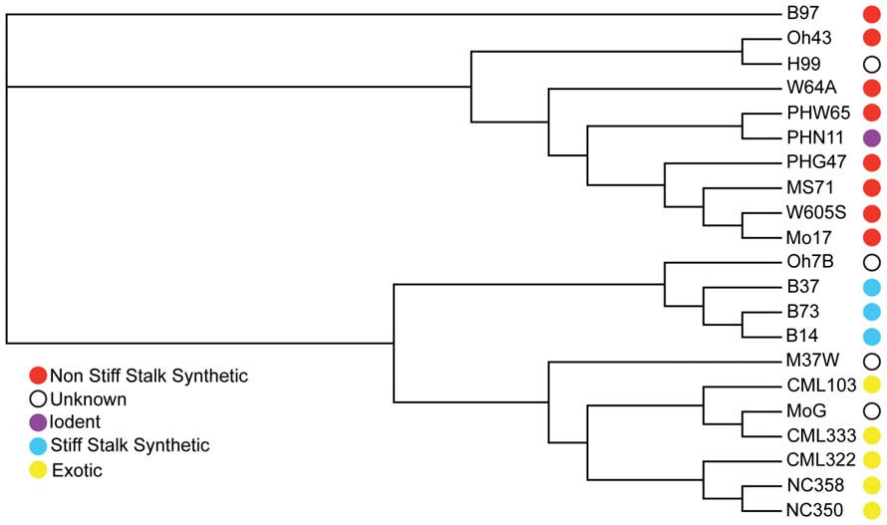
Neighbor-Joining tree of 21 diverse maize lines based on 351,710 single nucleotide polymorphisms (SNPs). Frequency based distances were calculated as pair-wise Rogers distances [53]. PowerMarker version 3.25 [54] was used to construct the tree.

Cluster analysis using allele frequency based distances from the 350,710 SNPs revealed the expected grouping of SSS and NSS type germplasm, and of exotic lines (Figure 2). Studies using 511 random SNP markers [8] and 100 simple sequence repeat markers [41] in larger diversity panels have also demonstrated that pedigree based groups cluster together using genomic markers. Higher marker density in this study markedly improved the clustering, although fewer lines were included.

### Transcriptome Profiles

In addition to the high allelic variation among these diverse lines, the transcriptome profiles were variable between the 21 inbred lines. The total number of expressed genes ranged from 22,522 to 26,026 (57.08% to 65.96%; Table 1), but the maximum observed expression value (fragments per kilobase of exon model per million fragments mapped, FPKM) varied substantially from 26,667 to 53,606 in M37W and PHW65, respectively (Table 1). In every inbred line except NC358, the gene with the maximum FPKM value encoded Chlorophyll a-b binding protein, as expected in photosynthetic seedling tissue.

Transcriptional variation on both a quantitative and qualitative level has been implicated in important agricultural traits [42]. Using a quantitative approach, we binned the genes as present or absent in the core maize seedling transcriptome. A gene was considered transcribed and included in the core transcriptome of a given inbred line if the FPKM 95% confidence interval lower boundary was greater than zero. Using this classification, 19,225 (48.7%) genes were expressed in all 21 inbred lines and 11,011 (27.9%) genes were expressed in one to 20 of the inbred lines in a fairly symmetric distribution (Figure S3). The 11,011 genes, which were expressed in only a subset of the lines, comprise the seedling variable transcriptome and may contribute to the phenotypic diversity observed in maize. However, sampling limitations and the range of sequence depth in this study are limitations in consistently detecting lowly expressed genes, and consequently, very lowly expressed genes may not be detected as expressed in a given genotype.

**Figure 3:**
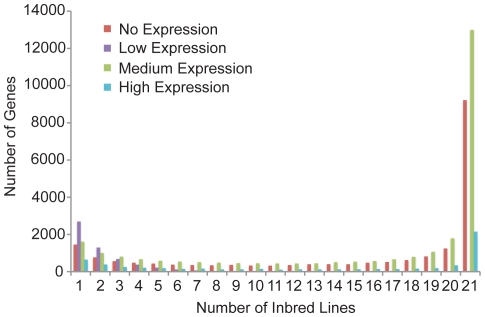
Distribution of genes in the maize seedling core and dispensable transcriptomes determined using a semi-qualitative approach. Reads were mapped to the 5b pseudomolecules (http://ftp.maizesequence.org/) using Bowtie version 0.12.7 [50] and TopHat version 1.2.0 [51], and fragments per kilobase of exon model per million fragments mapped (FPKM) were determined with Cufflinks version 0.9.3 [56] and the 5b annotation (http://ftp.maizesequence.org/).For each gene, a line was considered not expressed if the low confidence FPKM value was equal to zero, low expressed if the low confidence interval was greater than zero and the FPKM value was less than 5, medium expressed if the low confidence interval was greater than zero and the FPKM value was greater than or equal to 5 and less than or equal to 200, and high expressed if the low confidence interval was greater than zero and the FPKM value was greater than 200.

Transcriptome profile variation can extend beyond PAV for each transcript. Using a semi-quantitative approach where inbred lines were categorized as having no, low, medium, or high expression for each gene, we also observed variation in transcript abundance between the lines (Figure 3). In this classification approach, a gene could have constitutive expression across all 21 lines within any one of the four categories. Alternatively, a gene could have variable expression, with inbred lines categorized into multiple expression level categories. Using this method, the no, medium, and high expression categories had a similar distribution to that observed in the expressed/not expressed based analysis described above (Figure 3; Figure S3), where a large number of genes (24,378) had constitutive expression across all 21 inbred lines and the remainder of genes were variable in their expression across the lines. For the low expression category, the majority of the genes had only 5 or fewer lines with low expression, and the other lines were predominantly no or medium expression. A mere 12 genes had low expression in all 21 lines. It is possible that lowly expressed genes are less frequently expressed across all 21 genotypes, or that there is erroneous transcription in a small number of lines. However, this altered distribution is most likely a technical limitation attributable to sampling limitations. While there are a large number of genes with constitutive expression, there are also many genes with variable transcript abundance, both quantitatively and qualitatively that may contribute to observed phenotypic diversity.

### Novel Transcript Discovery and Presence/Absence Variation

Presence/absence variation on a genome level may be a contributing factor to the phenotypic diversity and heterosis observed in maize [16], [18], [19], [43], [44]. Whole genome array-based comparative genome hybridization experiments across 33 maize and teosinte lines have identified nearly four thousand genes exhibiting either CNV or PAV [18], [19]. Using 83.7 Gb of sequence across six elite inbreds related by pedigree and a combination of whole genome shotgun sequencing, mapping onto the B73 reference genome, and *de novo* assembly approaches, 296 genes in the B73 filtered gene set were identified as being absent in at least one of the non-B73 lines and at least 157 novel non-B73 genes were identified [16].

We used *de novo* assembly of the unmapped RNA-seq reads from all 21 lines to identify additional novel expressed genes that are either not present in the B73 inbred line or are missing from the B73 5b pseudomolecules altogether. Assembly of the unmapped RNA-seq reads using Velvet/Oases [45] with multiple k-mers indicated that 23 was the optimal k-mer (data not shown). The combined *de novo* assembly used 7,919,942 (17.5%) of the total unmapped reads to generate 8,595 transcripts across 4,701 loci, with an N50 of 725 bp. The average transcript size was 752 bp, with some transcripts greater than 3,000 bp (Figure S4). To identify truly novel sequences and remove those that were either alleles or paralogs with similar sequences in the B73 reference genome, the representative transcript for each locus was aligned to the B73 5b pseudomolecules. Multiple percent identity (Figure S5A) and percent coverage (Figure S5B) cutoffs were used to establish the optimal parameters for determining if a transcript should be retained. For both parameters, there was a large decrease in the number of transcripts that could be mapped between 85% and 90%, therefore the percent coverage and identity cutoffs were set to 85%. Using this filtering, 1,321 high confidence novel representative transcripts were retained, significantly more than previously identified using *de novo* assembly of genomic sequence [16], yet similar to that observed using comparative genome hybridization [18], [19]. Of these representative transcripts, 365 did not align to the 5b pseudomolecules at any alignment criteria, and 956 had alignments below the coverage and/or identity cutoff criteria. To confirm the novel nature of these transcripts, we re-mapped all of the RNA-seq reads from all 21 lines to an amended set of B73 5b pseudomolecules in which we added a supplemental pseudomolecule constructed by concatenating our novel representative transcripts. Using the same mapping parameters as described previously, the total number of mapped reads for every inbred line increased with the inclusion of the *de novo* assembled transcripts (Table 1), whereas the proportion of reads that mapped to multiple locations remained relatively constant (Figure S1).

RT-PCR was conducted on 21 of the filtered representative transcripts across eight inbred lines to confirm the predicted size and transcript PAV across the lines. The 21 transcripts were selected to represent transcripts with computationally predicted expression in one, four, or eight of the lines. The use of transcripts within these three categories allowed for more accurate experiment-wide inferences to be made. A transcript was predicted to be present in a given line if the FPKM 95% low confidence interval was greater than zero, and absent if the 95% confidence interval was equal to zero. Seven transcripts were concordant for both the *de novo* assembly length and PAV predictions. Another seven were concordant with the *de novo* assembly length but not with the FPKM predicted PAV, and the remaining seven transcripts did not amplify under the PCR conditions used (Table S3). In total, of the 14 transcripts that were amplified in the eight surveyed lines, only 14 of the 112 inbred line-transcript pairs (12.5%) did not match the computational PAV predictions. Additionally, all of the genes with predicted expression in each inbred line based on FPKM values were confirmed experimentally with RT-PCR. The lack of concordance in expression, i.e., positive experimentally with RT-PCR, but FPKM values equal to zero, are due to either insufficient sequence depth or sequence divergence resulting in the reads failing to map to the reference genome.

To further test the accuracy of the assembled transcript sequences, RT-PCR reactions with a visible band (75 reactions across the 14 novel transcripts) were Sanger sequenced from the 5′ and 3′ end of the fragment (Table S4). The assembled novel transcripts are the product of a hybrid assembly across the 21 maize lines. Because of this, we do not expect the assembly to represent a single inbred line in its entirety, but to be reflective of lines with higher expression levels, as reads from those lines will be more abundant. For all 14 novel transcripts, the sequence from the 5′ and 3′ ends contained complete coverage of the predicted sequence indicating that the assembled transcripts were likely not chimeras and that there were not large exons missing in the assembly. For all but one of the inbred line-transcript pairs, the percent identity for the bases that aligned was greater than 90%, indicating conservation of these gene sequences, while still reflecting some diversity between the lines. We also evaluated the percent coverage in each of the pair wise alignments between the assembled sequences and the Sanger sequences. For 12 of the 14 novel transcripts, the coverage was greater than 90% for all of the sequenced lines. The lower coverage for the remaining two transcripts is reflective of lower sequence conservation between the lines for some portion of the gene. The high validation rate of the RT-PCR and subsequent Sanger sequencing demonstrates the robust nature of the computational pipeline used to generate the *de novo* assembled transcripts and to predict presence/absence status of the transcript.

The novel transcripts that did not map to the B73 reference sequence may be the result of non-maize sequence contamination. To test for the presence of contamination, the high confidence assembled transcripts were searched against UniRef100 [46]. Of the 1,321 *de novo* assembled transcripts, 531 could be aligned to the UniRef100 database at greater than 70% coverage and 70% identity, and the top hit for these transcripts were all to *Poaceae* sequences. An additional 638 transcripts had alignments below this cutoff; 610 of these were to *Plantae* sequences and the remaining 28 were to non-*Plantae* sequences, indicating a very low contamination frequency. Similarly, alignments to the *Oryza sativa* and *Sorghum bicolor* protein sequences revealed 379 and 409 transcripts with alignments greater than 70% coverage and 70% identity, respectively, and 621 sequences with alignments below this cutoff to *Oryza sativa* and 616 to *Sorghum bicolor* protein sequences. Interestingly, only 225 transcripts of our *de novo* assembled transcripts aligned to the 181,717 maize PlantGDB-assembled Unique Transcripts (PUTs) [47] at greater than 85% coverage and 85% identity, and 214 of the transcripts did not align at all to the PUTs. This indicates that there is a breadth of maize sequence that has not yet been sampled.

**Figure 4:**
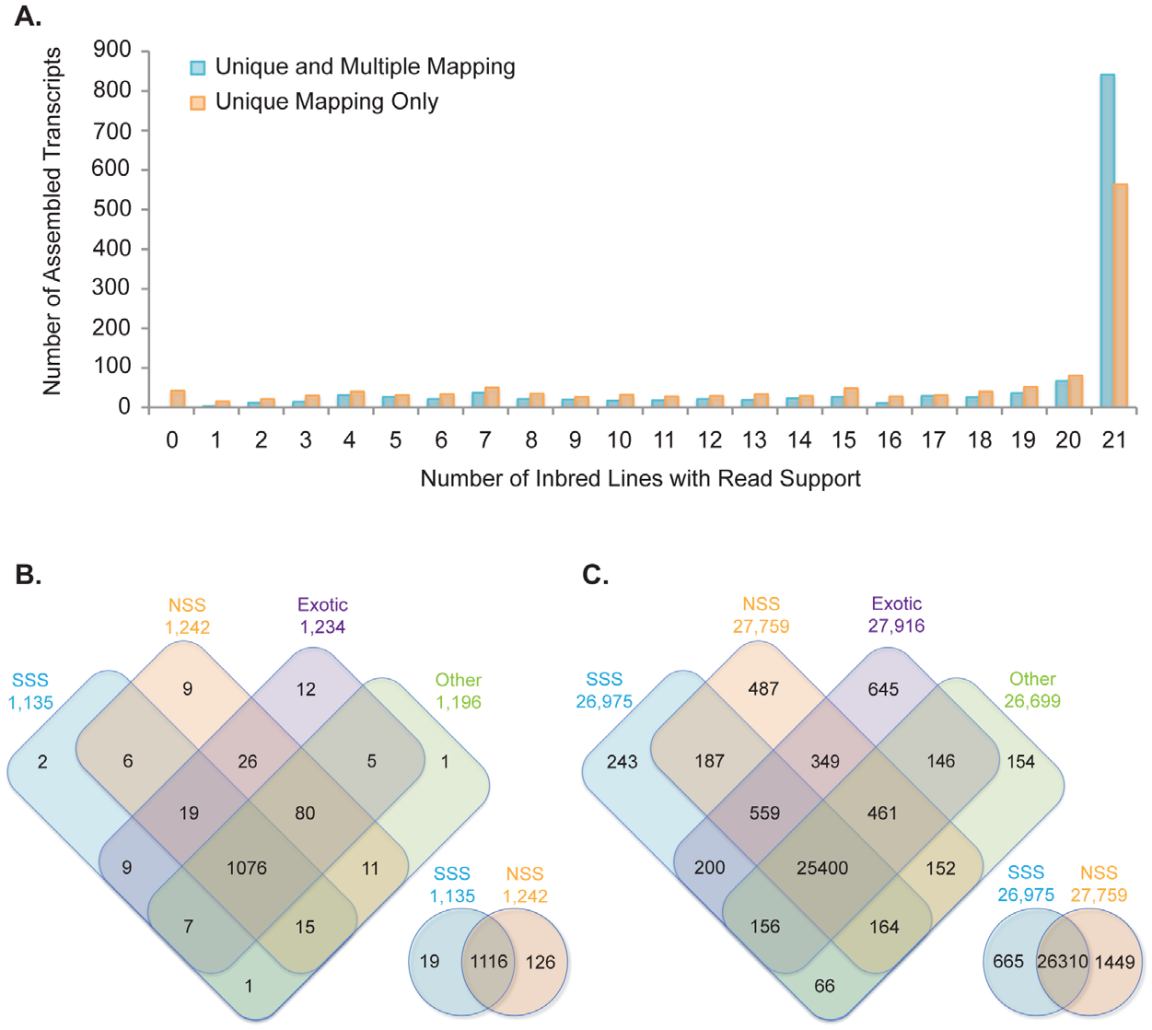
Frequency of novel *de novo* assembled transcripts across lines and heterotic groups. Reads were mapped to the 5b pseudomolecules plus assembled novel transcripts with Bowtie version 0.12.7 [50] and TopHat version 1.2.0 [51]. Novel transcripts from unmapped reads were assembled using Velvet version 1.0.17 and Oases version 0.1.18 [45]. (A) Distribution of the number of inbred lines with read support for each novel *de novo* assembled transcript requiring unique alignments and allowing for multiple mapping. (B) Venn diagram of shared and group specific novel *de novo* assembled transcripts. (C) Venn diagram of shared and group specific novel *de novo* assembled transcripts and transcripts from the 5b annotated genes (http://ftp.maizesequence.org/).

Across all 1,321 novel transcripts, a range in the number of lines with read support was observed (Figure 4A). Using a stringent criterion of at least one read mapping uniquely to the transcript, 42 transcripts across all 21 lines did not have support. However, reducing the stringency criterion such that reads were permitted to map to multiple locations, all of the *de novo* novel transcripts had read support. These transcripts with read support only when reads were allowed to map to multiple locations most likely represent paralogous genes or genes with CNV at a diverged locus, such that they cannot be aligned at greater than 85% coverage and 85% identity. A total of 564 transcripts had read support across all 21 lines and likely represent essential maize genes in the core genome that are missing from the current B73 reference assembly. There were 270 additional transcripts with read support in B73 yet missing in the current B73 reference assembly, but not present in one or more of the other lines. In total, when requiring reads to map uniquely, there were 715 transcripts with read support in one to 20 of the lines. These transcripts present in only a portion of the 21 lines are part of the dispensable transcriptome, and could be due to PAV on the genomic level or transcriptional variation due to promoter sequence variation or epiallelic variation [20], [21]. Of these 715 transcripts, 24 were specific to one of the pedigree groups, and comparison of only the SSS and NSS type germplasm, 145 were present in only one of the heterotic groups (Figure 4B). Expanding this to all genes in the 5b annotation and the assembled transcripts, 1,529 were specific to one of the pedigree groups (SSS, NSS, exotic, and other), and 2,114 were heterotic group specific in the SSS and NSS germplasm comparison (Figure 4C).

We have shown RNA-seq to be a robust, rapid, and inexpensive method to identify SNPs in genic regions in maize, an important crop species with a large, complex, repetitive genome. Furthermore, we have identified a core set of essential genes, as well as a set of genes that may be dispensable to the maize seedling transcriptome. Using *de novo* assembly, we have discovered transcripts previously unidentified in maize. We have also shown that the CNV and PAV observed at the genome level between lines in opposite heterotic groups extends to the transcriptome for the annotated maize genes as well as novel transcripts identified through assembly of RNA-seq within this set of inbred lines. In addition to these utilities, RNA-seq can also be used to analyze simple sequence repeat diversity in the gene space as well as the presence of short insertions/deletions using similar methods to those consistently used with genomic sequence. In contrast, due to the confounding of CNV in the genome and transcription rate variation, it is not practical to use RNA-seq data to infer CNV as can be determined with genomic sequence.

Heterosis, defined as the superior performance of F_1_ hybrids relative to their inbred parents, is a complex phenomenon that is likely underlined by multiple molecular mechanisms. This phenomenon has been highly exploited for economic gain through hybrid seed production, and in maize these gains are estimated to provide a 15% yield advantage to hybrids [48]. Understanding the molecular basis of heterosis can have major implications for improving yield beyond what has been realized to date with our limited understanding of the mechanisms underlying heterosis. It has been hypothesized that allelic complementation, structural variation, and epiallelic variation may contribute to this phenomenon [16], [18], [19], [20], [21], [43], [44]. The systematic divergence of germplasm by breeders for decades has generated relatively distinct heterotic groups within maize [36]. In this study, we showed tight clustering of genotypes within heterotic groups using genome wide SNP markers. It is likely that the heterosis observed from this allelic divergence is the product of complementation of deleterious alleles present within each heterotic group. Genomic level PAV between heterotic groups represents an extreme case of complementation (dominance model of heterosis) and has been shown in this study to extend to the transcriptome. The SSS and NSS heterotic groups in maize are large and diverse sets of germplasm, and because of such, it is necessary to test the extent to which the observations made in this study extend beyond these 21 inbred lines. While additional research is needed to definitively implicate allelic, structural, and transcriptome level variation in heterosis, this study provides growing evidence to the involvement of all of these levels of variation in heterosis.

## Materials and Methods

### Plant Materials and RNA Isolation

A set of 21 diverse maize inbred lines was evaluated in this study representing the SSS heterotic group (B14A, PI 550461; B37, PI 550467; B73, PI 550473), the NSS heterotic group (B97, PI 564682; Mo17, PI 558532; MS71, PI 587137; Oh43, Ames 19288; PHG47, PI 601318; PHW65, PI 601501; W605S, Ames 30557; W64A, NSL 30058), Iodent (PHN11, PI 601497), exotic (CML 103, Ames 27081; CML 322, Ames 27096; CML 333, Ames 27101; NC350, Ames 27171; and NC358, Ames 27175), and other (H99, PI 587129; M37W, Ames 27133; MoG, Ames 27136; and Oh7B, Ames 19323) germplasm. Plants were grown under greenhouse conditions (27°C/24°C day/night and 16 h/8 h light/dark) with five plants per pot (30 cm top diameter, 28 cm height, 14.5 L volume) in Metro-Mix 300 (Sun Gro Horticulture, http://www.sungro.com/) with no additional fertilizer. Whole above ground seedling tissue from three plants per inbred line was pooled 6–7 days after planting at the vegetative 1 stage [49], and immediately frozen in liquid nitrogen. Total RNA was extracted with TRIZOL (Invitrogen, http://www.invitrogen.com) and subsequently purified with the RNeasy MinElute Cleanup kit (Qiagen, http://www.qiagen.com), both according to the manufacturer’s protocol.

### RNA-seq Library Construction and Sequencing

mRNA was isolated from approximately 5 μg of total RNA, fragmented, converted to cDNA, and PCR amplified according to the Illumina RNA-seq protocol (Illumina, Inc. San Diego, CA, Cat # RS-100-0801), and sequenced using the Illumina Genome Analyzer II (San Diego, CA) at the University of Wisconsin Biotechnology Center (Madison, WI). Illumina barcodes were used to pool three samples per lane. Two technical replicates were conducted for each library to generate 36 and 74 bp single-end sequence reads. Sequences are available in the Sequence Read Archive at the National Center for Biotechnology Information (study accession number SRP006703). For all subsequent analysis, reads from the two technical replicates were pooled.

### Single Nucleotide Polymorphism Diversity Analysis

The FASTX toolkit (http://hannonlab.cshl.edu/fastx_toolkit/index.html) was used to clean reads prior to mapping. The fastx_clipper program was used to remove the Illumina adapter sequences requiring a minimum sequence length of 20 bp after clipping, and the fastq_quality_trimmer was used to remove low quality bases from the ends of reads requiring a minimum Phred score of 20 and a minimum length of 20 bp after trimming.

Cleaned RNA-seq reads were mapped to the maize B73 5b pseudomolecules (http://ftp.maizesequence.org/) [17] using Bowtie version 0.12.7 [50] and TopHat version 1.2.0 [51], requiring a minimum intron size of 5 bp and a maximum intron size of 60,000 bp. Alignments for reads that mapped uniquely to the pseudomolecules were processed using the sort, index, and pileup programs within SAMtools version 0.1.7 [52] to generate unfiltered pileup files. A custom Perl script was used to provide counts of reads for each nucleotide (A, T, C, and G) with a Phred score greater than 20 at each position. To determine the genotype for each of the inbred lines at each position, a nucleotide had to be present in greater than 5.0% of the filtered reads and a minimum of two of the filtered reads supporting that nucleotide to be included. A genotype containing more than one allele was defined as missing data as it is not possible to resolve if this is a true heterozygous locus or the product of a duplication not represented in the reference sequence with sequence divergence appearing heterozygous. To confirm the presence of an allele at each nucleotide position, we required data from at least two inbred lines to support the presence of that allele, and furthermore at least two alleles had to be present to call a SNP. Thus, with these criteria, SNPs could only be detected at a nucleotide position for which there was sequence coverage in at least four of the 21 genotypes. For example, to identify a biallelic SNP, data had to be present in at least two inbred lines for one allele and at least two other inbred lines for the alternative allele.

Genomic DNA sequence reads were obtained using genotyping-by-sequencing [39], with an additional size selection step to restrict the fragment size to approximately 300 bp. The multiplexed library was parsed into individual lines and barcode sequences removed using a custom Perl script that required a perfect match to the barcode and ApeKI cut site (GC[A/T]GC). The reads were cleaned using the same method as the RNA-seq reads and subsequently mapped to the 5b pseudomolecules using Bowtie version 0.12.7 [50] permitting up to two mismatches. The alignments were compiled using SAMtools version 0.1.7 [52] as described above. Genotype calls were determined with a custom perl script requiring at least 70% of the reads at a given loci to support a single nucleotide.

Functional annotation of genes with a high number of SNPs and high SNP density was obtained from maizesequence.org. Pair-wise allele frequency based distances between inbred lines were calculated as Rogers distances [53] using the 351,710 SNPs identified from the RNA-seq reads. PowerMarker version 3.25 [54] was used to construct the neighbor-joining tree, and TreeView version 1.6.6 was used to generate the tree image [55].

### Transcriptome Profile Analysis

To evaluate variation in gene expression, purity filtered reads were mapped to the 5b pseudomolecules (http://ftp.maizesequence.org/) using Bowtie version 0.12.7 [50] and TopHat version 1.2.0 [51], which utilize a quality and splice site aware alignment algorithm. The minimum and maximum intron length was set to 5 bp and 60,000 bp respectively; all other parameters were set to the default values. Normalized gene expression values were determined using Cufflinks version 0.9.3 [56], data provided in Table S5. The maximum intron length was set to 60,000 bp, the quartile normalization option was used, the 5b annotation (http://ftp.maizesequence.org/) was provided for the reference annotation, and the v2 pseduomolecules were provided for the bias detection and correction algorithms. The default settings were used for all other parameters.

The Cufflinks program provides the upper and lower bound FPKM values for a 95% confidence interval. For the qualitative categorization of genes based on expression status, if the low confidence interval value was greater than zero the gene was considered expressed. For the semi-quantitative categorization, a gene was considered lowly expressed if the low confidence interval was greater than zero and the FPKM value was less than 5, moderately expressed if the low confidence interval was greater than zero and the FPKM value was greater than or equal to 5 and less than or equal to 200, and highly expressed if the low confidence interval was greater than zero and the FPKM value was greater than 200.

### Identification and Characterization of Novel Maize Transcripts

RNA-seq reads were mapped to the 5b pseudomolecules (http://ftp.maizesequence.org/) using the same methods as described above for the transcriptome analysis. Unmapped reads were cleaned with the FASTX toolkit (http://hannonlab.cshl.edu/fastx_toolkit/index.html), using the fastx_clipper requiring a minimum read length of 30 bp after clipping, the fastx_artifacts_filter, and the fastq_quality_trimmer requiring a minimum Phred score of 20 and a minimum read length of 30 bp. Cleaned RNA-seq reads from all 21 lines were *de novo* assembled in a joint assembly using Velvet version 1.0.17 and Oases version 0.1.18 [45] requiring a minimum transcript length of 500 bp. Four assemblies were generated using a k-mer of 21, 23, 25, and 27. The optimal k-mer size based on the number of reads incorporated, number of loci/transcripts, and the N50 contig size was 23. For all future analyses only the representative transcript, defined as the longest transcript, for each locus was used. Representative transcripts were aligned to the 5b pseudomoleucles using GMAP version 2010-07-27 [57], a splice site aware aligner designed for mRNA and expressed sequence tag sequences. Transcripts with alignments greater than 85% coverage and 85% identity were removed from future analysis. The representative transcripts that did not align to the reference sequence were compiled into a single chromosome and added to the 5b pseudomolecules hereafter referred to as the reference plus assembly. All of the RNA-seq reads were aligned to the reference plus assembly and FPKM values determined using the methods described in the transcript analysis section. The reads were also aligned to the reference plus assembly requiring a unique alignment.

Primers were designed to confirm the transcript assembly and PAV for 21 representative transcripts from the assembly across eight of the inbred lines (B14A, B37, B73, B97, CML103, CML322, CML333, and H99; Table S3). The transcripts selected were computationally predicted to have expression in one, four, or eight of these lines. The same RNA used for the RNA-seq library construction was converted to cDNA using the SuperScript One-Step RT-PCR kit (Invitrogen, http://www.invitrogen.com) according to the manufacturer’s protocol and PCR amplified using the following program: 94°C for 15 sec, 58°C for 30 sec, and 70°C for 1.5 min, for 40 cycles, followed by 72°C for 8 min. RT-PCR products with a visible band were cleaned and prepared for sequencing using FastAP Thermosensitive Alkaline Phosphatase (Thermo Scientific, http://www.fermentas.com) according to the manufacturer’s suggested protocol. Sanger dideoxy-termination sequencing was performed at the Michigan State University Research Technology Support Facility (East Lansing, MI). Sequences were aligned to the computationally predicted sequences in pair-wise alignments with NCBI BLASTN [58] using the longest high scoring pair and in multiple sequence alignments with CodonCode Aligner version 3.7.1.1 (CodonCode Corporation, Dedham, MA, USA).

Assembled transcripts were searched against the UniRef100 database release 2011_04 [46] with WU BLASTX [59], [60] requiring a minimum E-value of 1e-5, a seed word length of 4, a neighborhood word score of 1000, and the shortqueryok option. The transcripts were also searched against the rice version 6.1 proteins [61] and the sorghum version 1 proteins [62] using WU BLASTX [59], [60], and against the maize PUTs excluding sequences less than 250 bases or with 10 or more N’s [47] using WU BLASTN [59], all requiring a minimum E-value of 1e-5. For all database searches only the best hit was used.

## Supporting Information

Figure S1 **Percentage of reads unmapped, mapped uniquely, and mapped multiple times.** Reads were mapped against the 5b pseudomolecules (http://ftp.maizesequence.org/) with Bowtie version 0.12.7 [50] and TopHat version 1.2.0 [51] and against the 5b pseudomolecules plus assembled transcripts representing novel sequences not present in the 5b reference pseudomolecules.
(TIF)

Figure S2 **Gene, gene expression, and single nucleotide polymorphism (SNP) density in 1** **Mb windows throughout the genome.** Number of genes was based on the 5b annotation (http://ftp.maizesequence.org/). RNA-seq reads were mapped to the genome with Bowtie version 0.12.7 [50] and TopHat version 1.2.0 [51] and fragments per kilobase of exon model per million fragments mapped (FPKM) were determined using Cufflinks version 0.9.3 [56] and the 5b annotation (http://ftp.maizesequence.org/). Four lines had to have a 95% confidence interval lower boundary greater than zero for a gene to be considered expressed, as this is the number of lines required for a SNP to be called. For SNP calling, a unique best hit was required.
(TIF)

Figure S3 **Distribution of genes in the maize seedling core and dispensable transcriptomes determined using a quantitative presence/absence classification.** Reads were mapped to the 5b pseudomolecules (http://ftp.maizesequence.org/) using Bowtie version 0.12.7 [50] and TopHat version 1.2.0 [51] and fragments per kilobase of exon model per million fragments mapped (FPKM) were determined using Cufflinks version 0.9.3 [56] and the 5b annotation (http://ftp.maizesequence.org/). A gene was considered expressed if the FPKM 95% low confidence interval boundary as defined by Cufflinks was greater than zero.
(TIF)

Figure S4 **RNA-seq** ***de novo*** **assembled transcript size distribution.** Reads that could not be mapped to the 5b pseudomolecules (http://ftp.maizesequence.org/) were *de novo* assembled with Velvet version 1.0.17 and Oases version 0.1.18 [45] requiring a minimum contig size of 500 bp.
(TIF)

Figure S5 **Summary of** ***de novo*** **assembled transcript mapping to the reference sequence at variable percent coverage and identity cutoffs.** The representative transcript for each locus, defined as the longest transcript, was mapped to the 5b pseudomolecules (http://ftp.maizesequence.org/) using GMAP [57] with coverage and identity cutoffs ranging from 70% to 95%. (A) Coverage constant and identity variable. (B) Coverage variable and identity constant.
(TIF)

Table S1 **Single Nucleotide Polymorphism (SNP) variants detected from RNA-seq in 21 diverse maize inbred lines.** Reads were mapped to the 5b pseudomolecules (http://ftp.maizesequence.org/) using Bowtie version 0.12.7 [50] and TopHat version 1.2.0 [51] requiring a unique alignment, and at least 2 reads and greater than 5.0% of the reads to support an allele.
(TXT)

Table S2 **Single nucleotide polymorphisms (SNPs) coverage across inbred lines and genes.** “Number of Inbred Lines” indicates the number of lines with sequence coverage in “Number of SNPs”. “Number of Genes” indicates how many genes are covered by at least one of these SNPs.
(XLSX)

Table S3 **Primers used for confirmation of the RNA-seq** ***de novo*** **assembly and expected transcript presence/absence variation.** Representative transcripts were selected for confirmation based on the predicted expression across 8 of the lines to represent transcripts with expression in 1, 4, or 8 of the lines.
(XLSX)

Table S4 **RT-PCR product Sanger sequencing summary.** RT-PCR products from reactions that had a visual band from 8 lines for 14 *de novo* assembled transcripts were Sanger sequenced and aligned to the computationally predicted sequences in pair-wise alignments with NCBI BLASTN [58] and in multiple sequence alignments with CodonCode Aligner version 3.7.1.1. Locus_1017_transcript_1 contained two non-overlapping high scoring pairs that were both included, for all other transcripts a single longest high scoring pair was used.
(XLSX)

Table S5 **Fragments per kilobase of exon model per million fragments mapped (FPKM) for seedling tissue from 21 diverse maize inbred lines.** Reads were mapped to the 5b pseudomolecules (http://ftp.maizesequence.org/) using Bowtie version 0.12.7 [50] and TopHat version 1.2.0 [51] and fragments per kilobase of exon model per million fragments mapped (FPKM) were determined using Cufflinks version 0.9.3 [56] and the 5b annotation (http://ftp.maizesequence.org/).
(XLSX)
