# Supplementary figures and images for: Maize (Zea mays L.) Genome Diversity as Revealed by RNA-Sequencing
Source: PLoS One. 2012 Mar 16;7(3):e33071. doi: 10.1371/journal.pone.0033071 (PMC3306378; doi:10.1371/journal.pone.0033071)

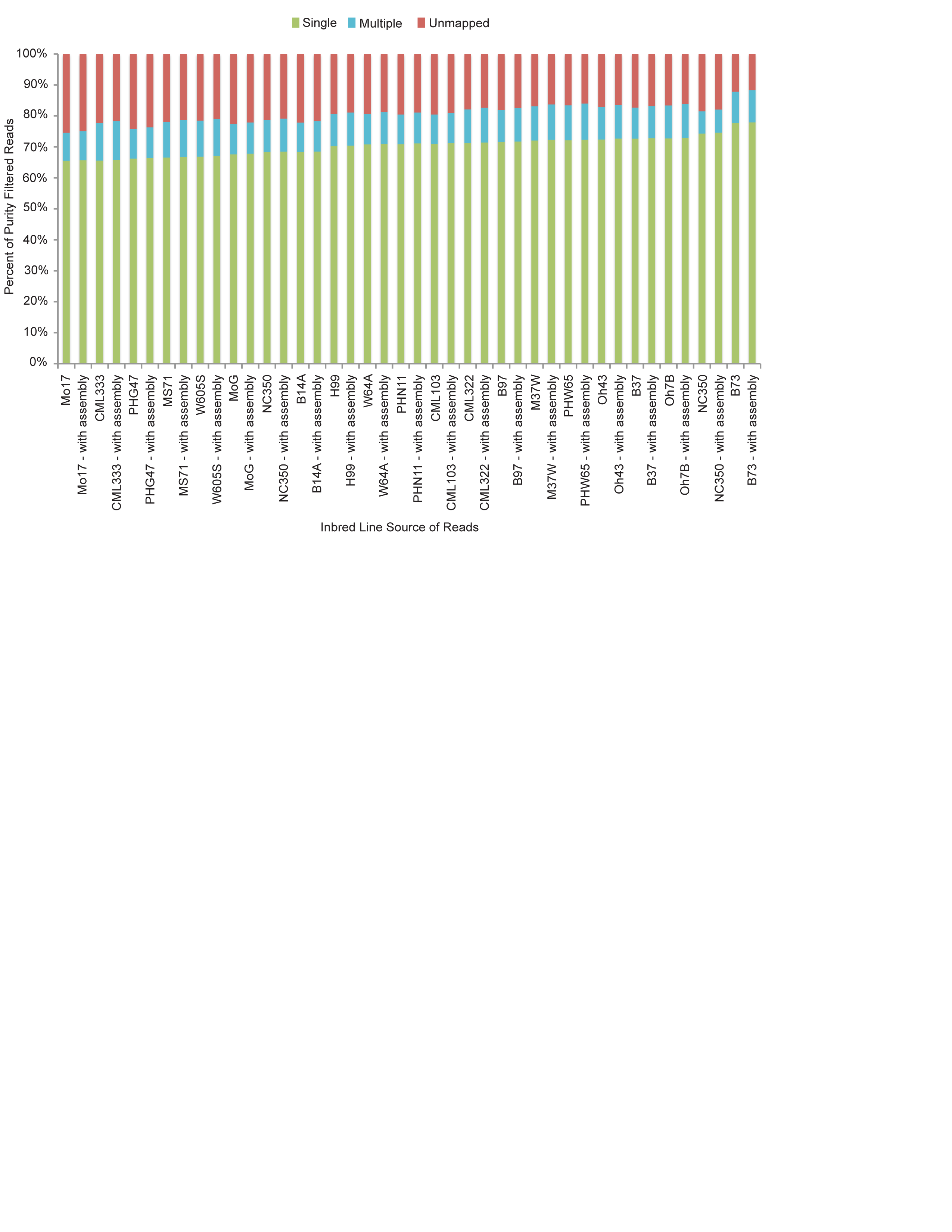

Supplement: Figure S1 — Percentage of reads unmapped, mapped uniquely, and mapped multiple times. Reads were mapped against the 5b pseudomolecules (http://ftp.maizesequence.org/) with Bowtie version 0.12.7 [50] and TopHat version 1.2.0 [51] and against the 5b pseudomolecules plus assembled transcripts representing novel sequences not present in the 5b reference pseudomolecules. (TIF) [file pone.0033071.s001.tif]

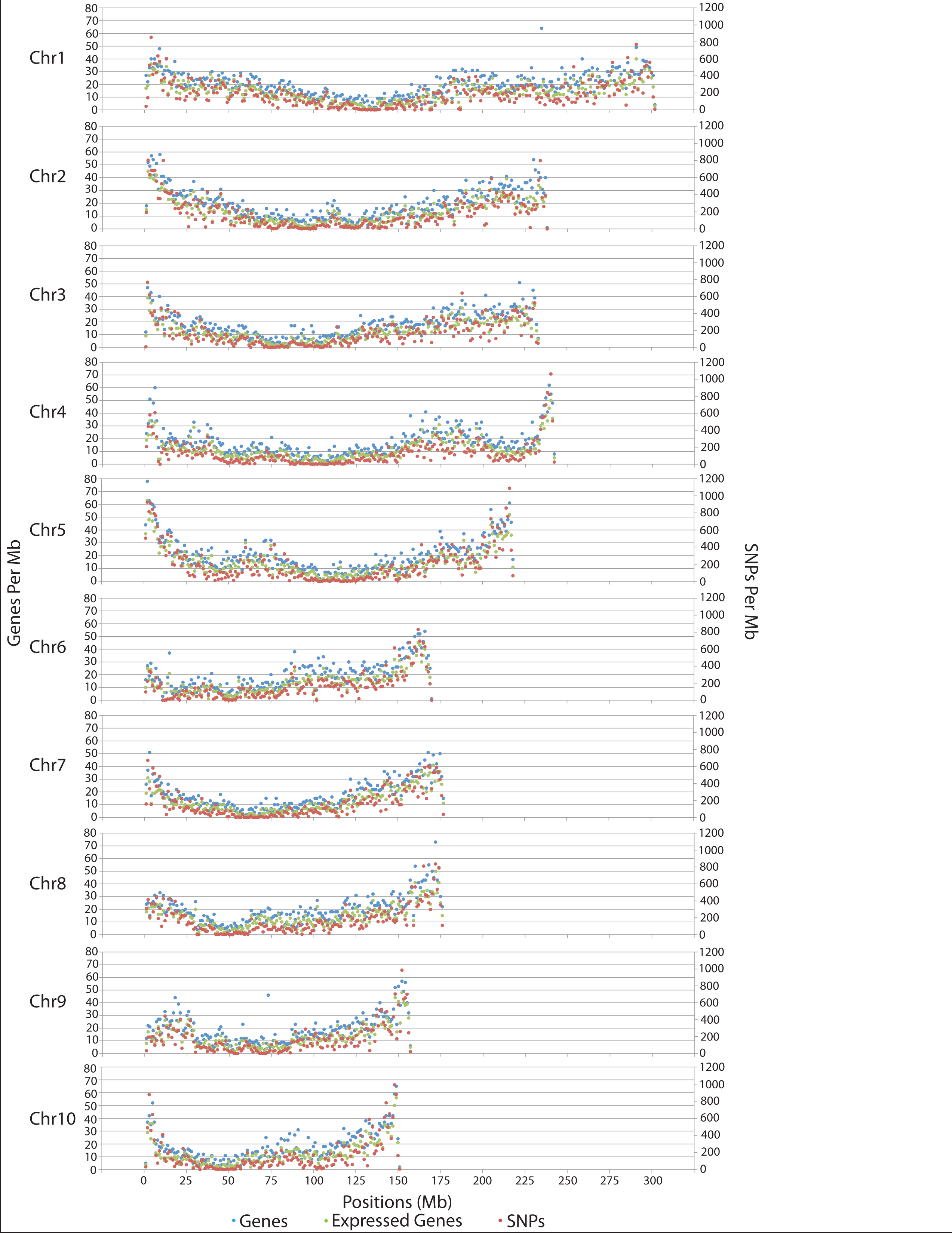

Supplement: Figure S2 — Gene, gene expression, and single nucleotide polymorphism (SNP) density in 1 Mb windows throughout the genome. Number of genes was based on the 5b annotation (http://ftp.maizesequence.org/). RNA-seq reads were mapped to the genome with Bowtie version 0.12.7 [50] and TopHat version 1.2.0 [51] and fragments per kilobase of exon model per million fragments mapped (FPKM) were determined using Cufflinks version 0.9.3 [56] and the 5b annotation (http://ftp.maizesequence.org/). Four lines had to have a 95% confidence interval lower boundary greater than zero for a gene to be considered expressed, as this is the number of lines required for a SNP to be called. For SNP calling, a unique best hit was required. (TIF) [file pone.0033071.s002.tif]

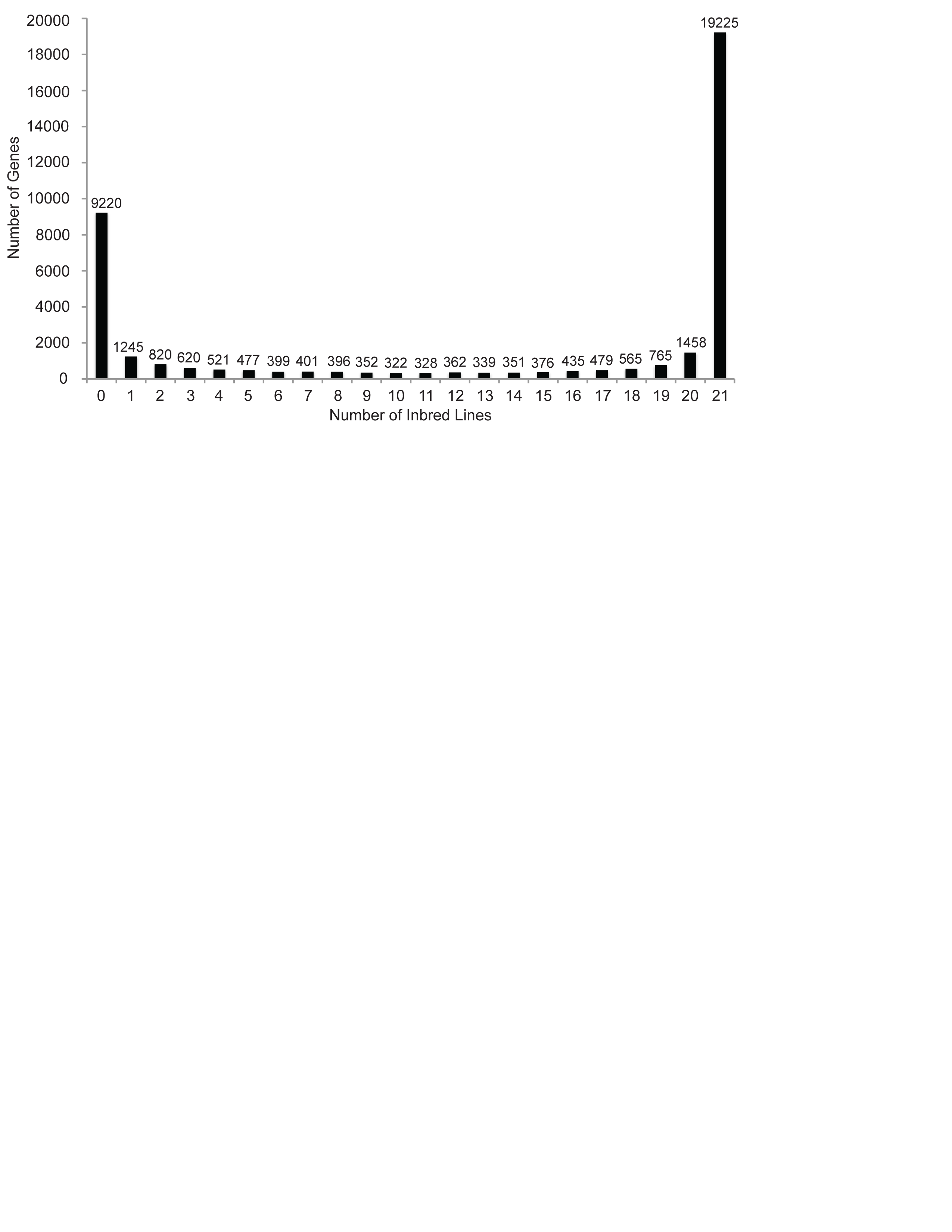

Supplement: Figure S3 — Distribution of genes in the maize seedling core and dispensable transcriptomes determined using a quantitative presence/absence classification. Reads were mapped to the 5b pseudomolecules (http://ftp.maizesequence.org/) using Bowtie version 0.12.7 [50] and TopHat version 1.2.0 [51] and fragments per kilobase of exon model per million fragments mapped (FPKM) were determined using Cufflinks version 0.9.3 [56] and the 5b annotation (http://ftp.maizesequence.org/). A gene was considered expressed if the FPKM 95% low confidence interval boundary as defined by Cufflinks was greater than zero. (TIF) [file pone.0033071.s003.tif]

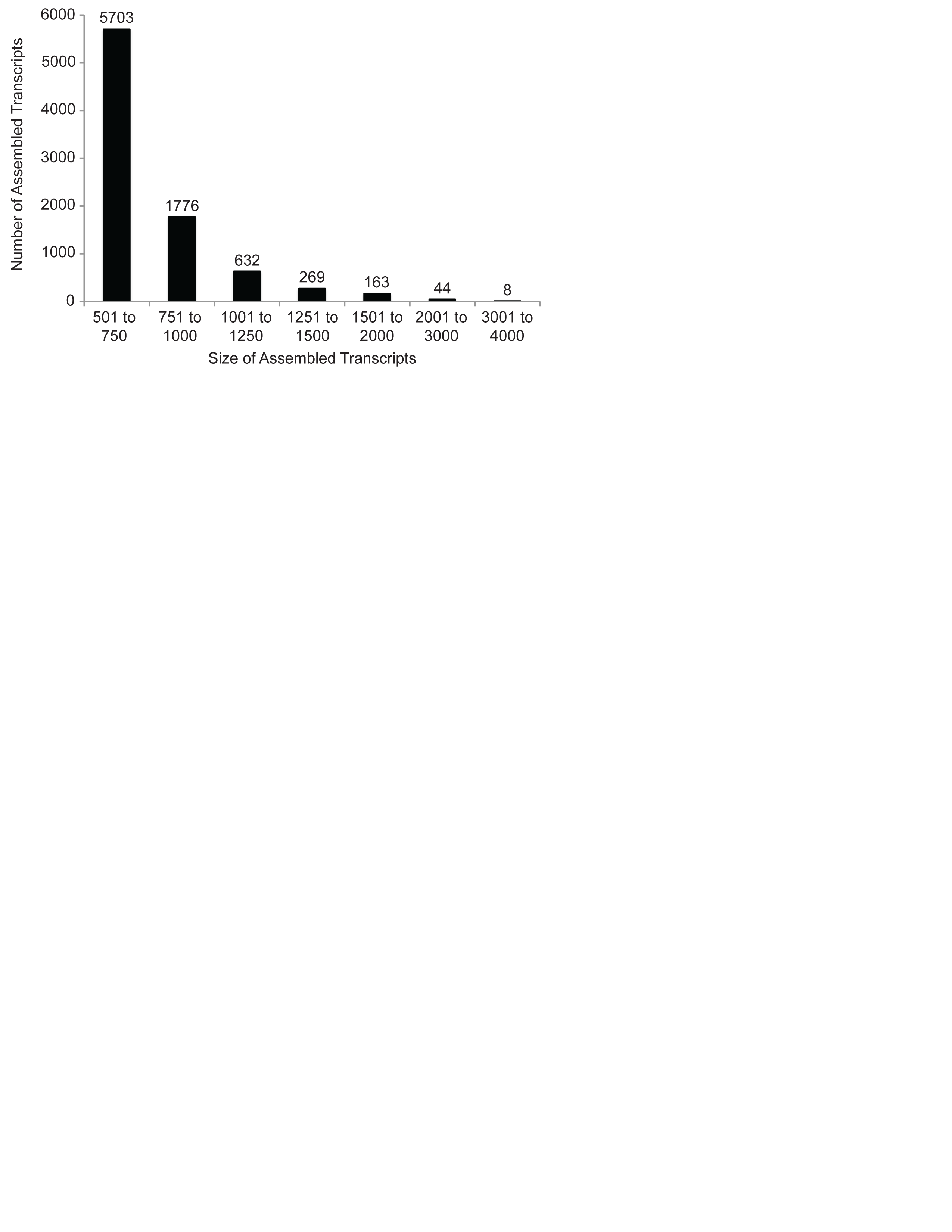

Supplement: Figure S4 — RNA-seq de novo assembled transcript size distribution. Reads that could not be mapped to the 5b pseudomolecules (http://ftp.maizesequence.org/) were de novo assembled with Velvet version 1.0.17 and Oases version 0.1.18 [45] requiring a minimum contig size of 500 bp. (TIF) [file pone.0033071.s004.tif]

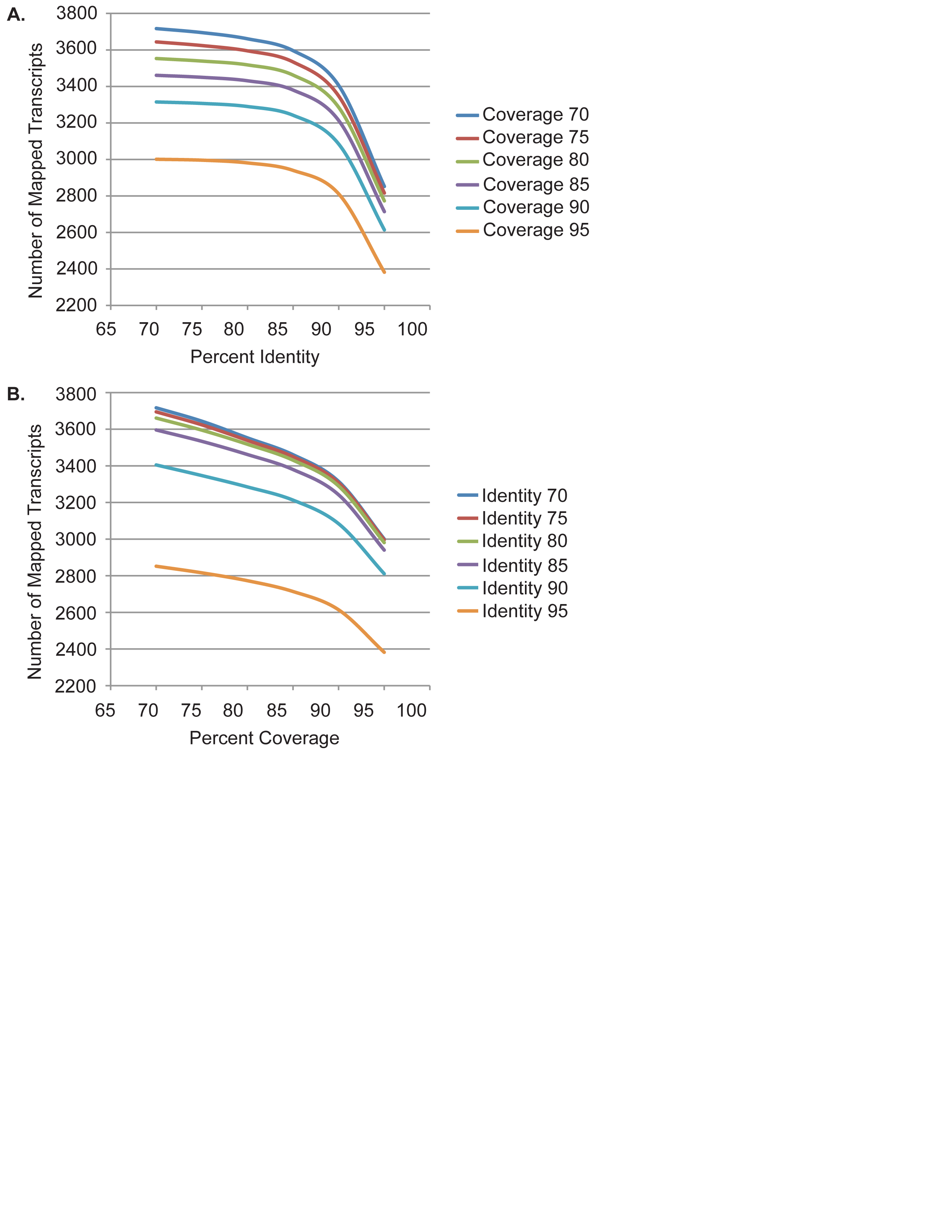

Supplement: Figure S5 — Summary of de novo assembled transcript mapping to the reference sequence at variable percent coverage and identity cutoffs. The representative transcript for each locus, defined as the longest transcript, was mapped to the 5b pseudomolecules (http://ftp.maizesequence.org/) using GMAP [57] with coverage and identity cutoffs ranging from 70% to 95%. (A) Coverage constant and identity variable. (B) Coverage variable and identity constant. (TIF) [file pone.0033071.s005.tif]
